# Prediction of spherical equivalent difference before and after cycloplegia in school-age children with machine learning algorithms

**DOI:** 10.3389/fpubh.2023.1096330

**Published:** 2023-04-11

**Authors:** Bei Du, Qingxin Wang, Yuan Luo, Nan Jin, Hua Rong, Xilian Wang, Hong Nian, Li Guo, Meng Liang, Ruihua Wei

**Affiliations:** ^1^Tianjin Key Laboratory of Retinal Functions and Diseases, Tianjin Branch of National Clinical Research Center for Ocular Disease, Eye Institute and School of Optometry, Tianjin Medical University Eye Hospital, Tianjin, China; ^2^School of Medical Technology, Tianjin Medical University, Tianjin, China; ^3^Tianjin Beichen Traditional Chinese Medicine Hospital, Tianjin, China

**Keywords:** cycloplegia, children, machine learning, refractive error, refractive state

## Abstract

**Purpose:**

To predict the need for cycloplegic assessment, as well as refractive state under cycloplegia, based on non-cycloplegic ocular parameters in school-age children.

**Design:**

Random cluster sampling.

**Methods:**

The cross-sectional study was conducted from December 2018 to January 2019. Random cluster sampling was used to select 2,467 students aged 6–18 years. All participants were from primary school, middle school and high school. Visual acuity, optical biometry, intraocular pressure, accommodation lag, gaze deviation in primary position, non-cycloplegic and cycloplegic autorefraction were conducted. A binary classification model and a three-way classification model were established to predict the necessity of cycloplegia and the refractive status, respectively. A regression model was also developed to predict the refractive error using machine learning algorithms.

**Results:**

The accuracy of the model recognizing requirement of cycloplegia was 68.5–77.0% and the AUC was 0.762–0.833. The model for prediction of SE had performances of R^2 0.889–0.927, MSE 0.250–0.380, MAE 0.372–0.436 and r 0.943–0.963. As the prediction of refractive error status, the accuracy and F1 score was 80.3–81.7% and 0.757–0.775, respectively. There was no statistical difference between the distribution of refractive status predicted by the machine learning models and the one obtained under cycloplegic conditions in school-age students.

**Conclusion:**

Based on big data acquisition and machine learning techniques, the difference before and after cycloplegia can be effectively predicted in school-age children. This study provides a theoretical basis and supporting evidence for the epidemiological study of myopia and the accurate analysis of vision screening data and optometry services.

## Introduction

Several studies have suggested that cycloplegic refraction should be considered the gold standard for epidemiological studies on refraction in school-aged children (1–3). Non-cycloplegic refractions are prone to significant errors, largely due to an active accommodation response (4–8). However, cycloplegia is challenging to perform for vision screening and epidemiological studies, resulting in a biased classification of ametropia; in fact, accommodation response cause a more negative value in SE, overestimating the presence and severity of myopia, and underestimating that of hyperopia (9, 10). Due to these biases, the research on myopia risk factors is likely to be significantly misguided, and inter-study comparisons will be affected (11–13). Therefore, an accurate prediction of SE after cycloplegia based on non-cycloplegic data could be an effective way to improve the accuracy of data in large-scale screening and epidemiological studies and would also be suitable for children for whom cycloplegia is contraindicated or refused.

Previous studies have examined the correlation between the difference in refraction before and after cycloplegia and patient characteristics. Significant correlations were found between age, non-cycloplegic spherical equivalent (SE), cylindrical power, intraocular pressure, whether wearing glasses and Lag of accommodation, axial length (14–16). A number of studies have tried to establish a prediction model based on the above-mentioned characteristics to predict the need for cycloplegia in children and adolescents. However, there are some problems in the research, such as the lack of independent validation set, insufficient number of features, simple mode, and the efficiency of prediction model is not ideal (13, 15, 16).

In the present study, detailed demographic and other relevant personal information, as well as vision screening data, of 2,467 school-age children were collected to explore the key influencing SE changes before and after cycloplegia. Our study could provide a convenient and helpful cycloplegic SE prediction model for clinical and epidemiological research.

## Materials and methods

### Ophthalmic examination and data set establishment

Data from 4,934 eyes of 2,467 school-age students was collected by Tianjin Medical University Eye Hospital in China during December 2018 to January 2019. None of the participants had a history of ocular disease or surgery. Written informed consent was obtained from parents or guardians, and verbal consent from all participants. This study was approved by the Institutional Ethics Committee of Tianjin Medical University Eye Hospital and followed the tenets of the Declaration of Helsinki for human research.

All subjects underwent general ocular examinations including visual acuity, non-cycloplegic autorefraction, optical biometry measurement, non-contact tonometry, lag of accommodation and gaze deviation in the primary position, which are basically routine examinations for school-age children and the digital results can be obtained directly to facilitate data processing. Subjects were excluded if their intraocular pressure was >25 mmHg. Cycloplegia was induced by instilling three drops of 1% cyclopentolate at 5 min intervals in each eye. One more drop of 1% cyclopentolate was administered if pupillary light reflex was still present or the pupil size was less than 6.0 mm at 30 min after the last drop. Cycloplegic refraction of both eyes was measured using the same autorefractor.

Visual acuity (uncorrected or with habitual correction, if any) was determined using a mounted and illuminated E chart at 5 m with ambient room lighting. Non-cycloplegic and cycloplegic refraction were measured with an autorefractor (KR.800, Topcon, Tokyo, Japan). Three repeated measurements were taken and averaged. The differences between the three readings had to be within 0.50D in both the spherical and cylinder components. Optical biometry parameters were measured using LENSTAR LS 900 system (HAAG-STREIT, Mason, Switzerland), including axial length, central corneal thickness (CCT), anterior chamber depth, lens thickness, and flat and steep keratometry. An average of the three measurements was considered in further analysis. If any two measurements of axial length differed by ≥0.02 mm, the readings were discarded and the eye remeasured. Intraocular pressure (IOP) was assessed using non-contact tonometer (CT.1 computerized tonometer, Topcon, Tokyo, Japan). Lag of accommodation (LOA) was measured by the open-field binocular autorefractor/keratometer (WR-5100 K; Grand Seiko Co., Ltd., Hiroshima, Japan). The subjects were instructed to view 20/100 Snellen letter at a distance of 33 cm, wearing the trial frame, with one eye occluded. Three repeated examinations were conducted, and the average SE was recorded as the accommodative response when the difference between the maximum and minimum was <0.25 D. LOA was calculated by subtracting accommodative response from accommodative stimulus (3.00 D). Gaze deviation in the primary position was measured using a Spot Vision Screener. Basic information including age, gender, and whether wearing glasses was collected by questionnaire.

**Figure 1:**
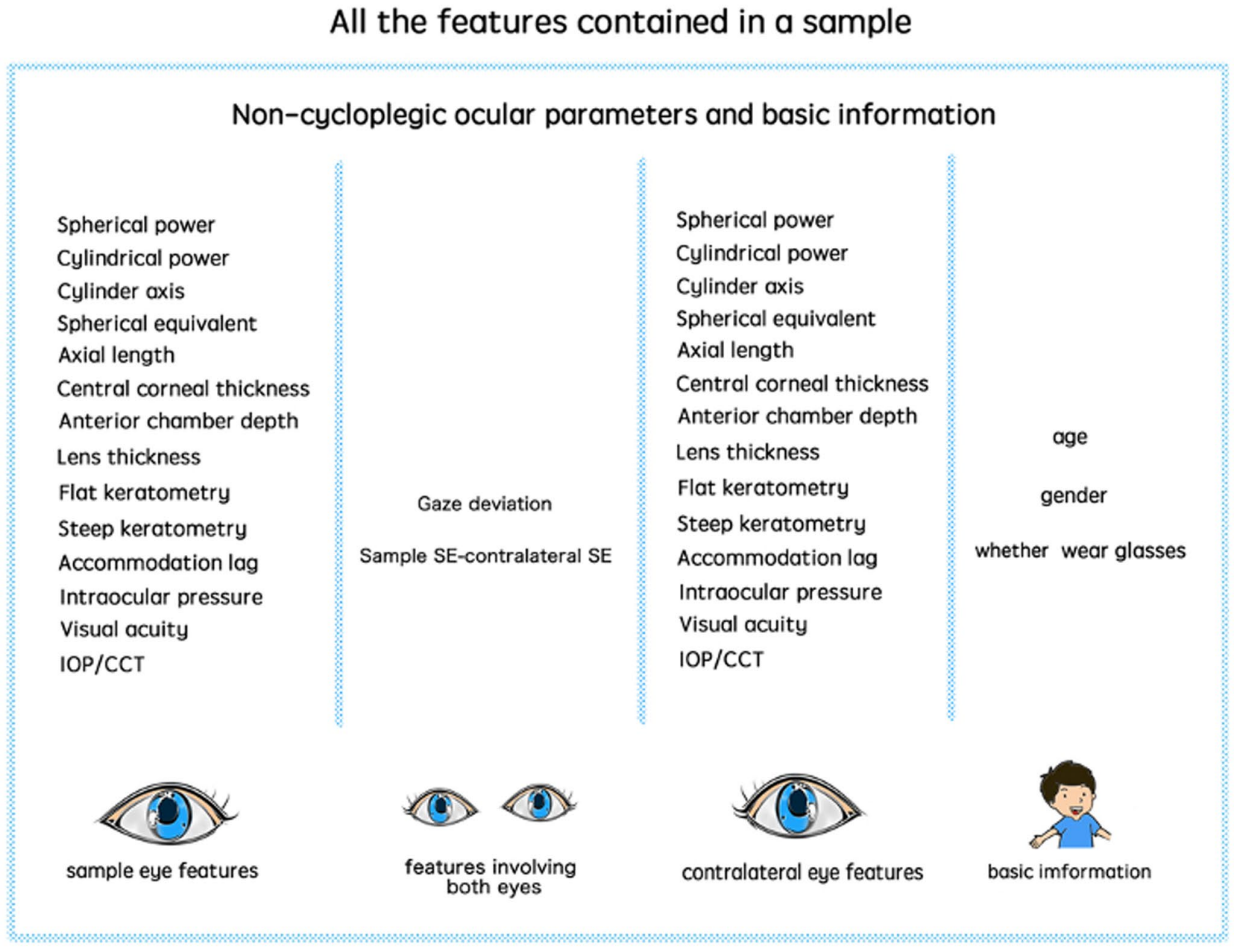
All the features contained in a sample.

A total of 33 parameters were included in the model, including 3 combined features (Figure 1). For the three combined features, one was the difference in SE between the sample-eye and the contralateral-eye (sample eye SE-contalateral eye SE), in order to avoid the effects of continuous adjustment fluctuations caused by binocular diopter disparity (17); the other two combined parameters were sample IOP/CCT and contralateral IOP/CCT, because the corneal thickness may affect the measurement of intraocular pressure (18).

Some values (4.6%) were missing in the non-cycloplegic ocular data due to technical problems, and we used a multivariate imputation method performed in an iterative round-robin fashion (19). More specifically, Bayesian ridge regression model was used to establish a mapping between each feature with missing values and other features to make predictions of missing data for each feature one by one, starting from the features with the least missing value (20). The final imputed data were obtained after iterating this imputation process for many rounds. In the present study, this imputation procedure was carried out using the Iterative Imputer algorithm implemented in the scikit-learn library and the iteration number was set to 100 (21).

We established prediction models at three outcome levels (online Supplementary Figure S1), each for a different clinical or epidemiological need in real practices. First, a binary classification model was trained to predict whether the subject had significant differences between cycloplegia and non-cycloplegia refractive measurement. Second, a three-way classification model was trained to predict cycloplegic refractive status. Third, a regression model was trained to predict cycloplegic refractive error.

### Predicting the need for cycloplegia

In order to predict whether a child needs cycloplegia to obtain accurate refraction data, we performed a classification analysis to distinguish the eye samples with large differences in refractive error before and after cycloplegia (i.e., those who need cycloplegic) and those with small differences (i.e., those who do not need cycloplegic).

We tested three different thresholds to define large vs. small differences in refractive error (calculated as the value of cycloplegic SE minus non-cycloplegic SE), each corresponding to different needs of clinical and research tasks: 0.25, 0.50, and 0.75 D.

Binary classification models were trained and tested for each of the three thresholds using the following procedure: (1) 10% of all participations were randomly selected with both their eyes as the independent test dataset (i.e., 492 eyes) and the remaining 90% (i.e., 4,442 eyes) as the training dataset, (2) For the training set, the raw values of each feature was normalized to Z scores (i.e., mean of 0 and standard deviation of 1) using the following equation: $z_{i}=\left(x_{i}-{\bar{x}}_{i}\right)/s_{i}\;$ , where $x_{i}$ is the i-th feature vector, and ${\bar{x}}_{i}\;$ and $s_{i}$ are the mean and standard deviation of ${\bar{x}}_{i}$ , respectively. For the test set, each feature’s mean and standard deviation values of the training dataset (i.e., the above ${\bar{x}}_{i}$ and $s_{i}$ ) were used to normalize the corresponding features of the test set, (3) During model training, 10-fold cross-validation within the training set was used for optimizing hyperparameters with the Hyperopt package (22, 23). Hyperopt is a Bayesian optimization method using a continuously updated probabilistic model based on hyperparameters and validation losses, which allows the search process to focus more on the hyperparameters that are likely to be optimal by reasoning from past validation losses, (4) Once the optimal hyperparameters were determined, the final model was trained using the full training set and evaluated using the test set, and (5) The performance of the final model was assessed using the receiver operating characteristic curve (ROC), classification accuracy, area under the ROC curve (AUC), sensitivity, and specificity.

Using the above machine learning procedure, we tested four machine learning algorithms: support vector machine (SVM) (24, 25), Random forest (RF) (26), Deep Neural Network (DNN) (27, 28), and Easy Ensemble Classifier (EEC) (29). Note that, at each threshold for defining the class labels of the eye samples (i.e., samples with large vs. small refractive error differences) in the present study, the number of samples of the two classes were unbalanced, which would lead to biased classification results. Therefore, we also adopted some balancing strategies for each machine learning algorithm to tackle this problem during model training, as follows:

1. SVM aims to find a decision hyperplane with a maximal margin separating the samples of two classes. To deal with the problem of unbalanced samples, we corrected the decision hyperplane by adjusting the parameter $C_{i}$ for Class *i* with a weight: $C_{i}=classweight_{i}\times C$ , where $classweight_{i}=\left(\underset{i}{\sum }n_{i}\right)/\left(2\times n_{i}\right)$ and $n_{i}$ is the sample size of Class *i* in the training dataset.
2. RF builds many decision tree models as the base learner, randomly samples a subset of features and a subset of training samples to train each decision tree, and then ensembles the results of all decision trees to form the final classification result based on the strategy of Bagging (30, 31).To deal with the problem of unbalanced samples, impurity calculations and prediction voting were adjusted using the same class weight as used in SVM.
3. DNN uses multiple fully connected layers and a non-linear activation function after each hidden layer to learn the feature representation of the nature of the original data, thereby facilitating the classification. To deal with the problem of unbalanced samples, different weights were assigned to the losses calculated for different classes during the training process so that the feedback given by the two classes were comparable when the error was back-propagated.
4. EEC is a classifier ensemble algorithm, specifically designed for learning with unbalanced samples. It draws a subset of samples from the majority class by bootstrapping to form balanced subsets of samples between the two classes for training an AdaBoost classification model (32). By repeating this procedure many times, multiple AdaBoost models were trained, and the classification results of all models were aggregated to form the final classification result.

### Predicting the cycloplegic refractive state

To predict the cycloplegic refractive status using non-cycloplegic ocular parameters, each eye sample was categorized into three groups according to the cycloplegic SE – myopia (SE ≤ -0.50D), emmetropia (-0.50D < SE < 0.50D) and hyperopia (SE ≥ 0.50D). The same machine learning algorithms and classification procedures as described above were used here, except that a multi-class classification rather than a binary classification problem was to be solved. The confusion matrix, accuracy (ACC), precision, recall and F1 score were used to assess the classification performance. As a control condition, all eye samples were also categorized into the three refractive states mentioned above based on the non-cycloplegic SE. By comparing the refractive state predicted by the machine learning models with those defined directly using non-cycloplegic SE, we could assess how much the machine-learning-derived results could improve the accuracy of the non-cycloplegic-SE-defined refractive status.

### Predicting the cycloplegic refractive error

As the cycloplegic refractive error has continuous values, predicting the actual values of cycloplegic refractive error corresponds to solving a regression problem, rather than a classification problem. Here，we tested four machine-learning algorithms for regression: Support Vector Regression (SVR), Random Forest Regression (RFR), AdaBoost Regression (ABR), and DNN. The training and test datasets were created in the same way as described in the above classification task, and the hyperparameters were optimized using the same 10-fold cross-validation procedure during training. The performance of the prediction model was assessed using *r*^2^, *r*, mean absolute error (MAE), mean squared error (MSE), as well as the proportion of the samples with small prediction errors (< 0.50 D). The predicted SE and the true cycloplegic SE were also statistically compared using matched T-test to test whether there was a significant difference between them. To test whether the predicted SEs were significantly closer, than the non-cycloplegic SE, to the true cycloplegic SEs, the *r*^2^, *r*, MAE, MSE, as well as the proportion of the samples with small prediction errors were calculated to assess the fitting degree between the cycloplegic SE and the non-cycloplegic SE. The *T*-test was also used to test whether there was a significant difference between them.

## Results

### Basic refractive results

A total of 4,934 eye samples of 2,467 children were included in this study and the mean age was 8.92 ± 2.21 years (ranging from 6 to18 years) and 1,292 participants (52.4%) were males. Before cycloplegia, the mean value of SE was −1.13 ± 1.58D and the prevalence was 60.3, 30.8 and 8.9% for myopia, emmetropia and hyperopia, respectively. After cycloplegia, the mean value of SE was −0.51 ± 1.81D, with a mean difference of 0.63 ± 0.69D compared with the non-cycloplegic SE, and the prevalence changed to 43.2, 21.7, and 35.1% for myopia, emmetropia and hyperopia, respectively.

### Prediction of the need for cycloplegia

When the threshold for defining large and small refractive SE changes was set to 0.25 D, the positive (i.e., the samples with large SE changes) in the training set and test set accounted for 66.1 and 67.3%, respectively. With the threshold was set to of 0.50D, the proportions of the positive samples in the training and test sets were 47.0 and 44.5%, respectively. When the threshold was set to 0.75D, the proportions of the positive samples in the training and test sets were 30.6 and 31.1%, respectively.

**Figure 2:**
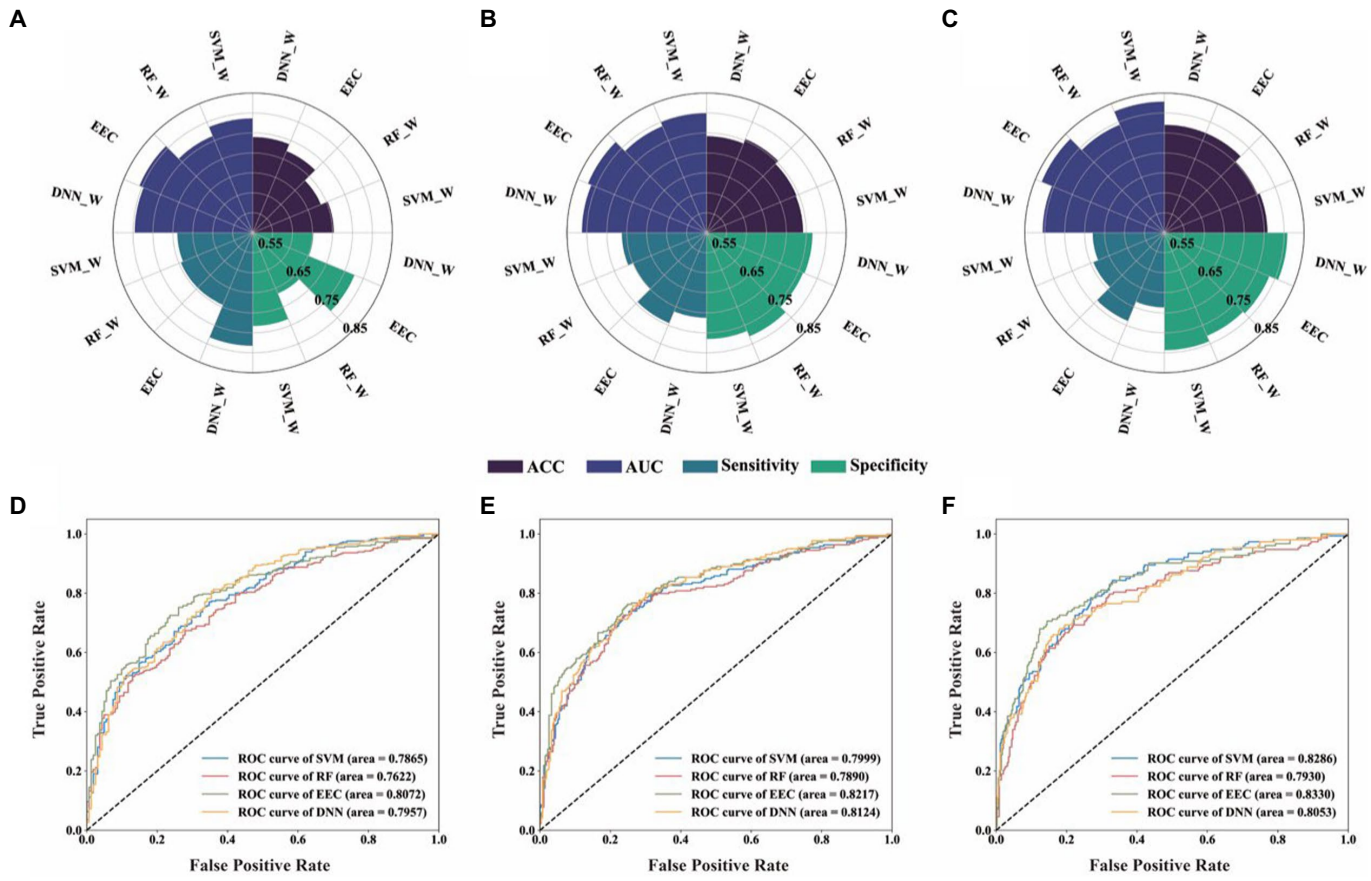
Performance of four machine learning models for binary classification of each class defining threshold. **(A**,**D)**, Threshold 0.25D, diagnostic values and ROC analysis of the four different predictive model; **(B**,**E)**, Threshold 0.5D, diagnostic values and ROC analysis of the four different predictive model; **(C**,**F)**, Threshold 0.75D, diagnostic values and ROC analysis of the four different predictive model. SVC_W, SVC algorithm with balancing strategies; RF_W, RF algorithm with balancing strategies; DNN_W, DNN algorithm with balancing strategies. (SVM_W, Support Vector Machine with balance method; RF_W, Random Forest with balance method; DNN_W, Deep Neural Network with balance method; EEC, Easy Ensemble Classifier; ROC curve, receiver operating characteristic curve; ACC, accuracy; AUC, area under the ROC curve).

The performances of the “large vs. small SE changes” classification obtained using the four machine learning algorithms for each class defining threshold were summarized in online Supplementary Table S1 and Figure 2. For contrast, the results obtained without sample balancing strategies are shown in online Supplementary Table S2 and online Supplementary Figure S2. The results confirmed that the machine learning algorithms performed much better when the sample balancing strategies were adopted: At the class defining threshold of 0.25D, very low specificities (49.7–54.0%) were obtained when the sample balancing strategies were not adopted as there were more positive samples than negative samples, and they were much improved (65.2–77.7%) when the sample balancing strategies were adopted; at the threshold of 0.75 D, very low sensitivities (47.1–60.1%) were obtained when the sample balancing strategies were not adopted as there were fewer positive samples than negative samples, and they were much improved (68.0–73.9%) when the sample balancing strategies were adopted.

Regarding the results obtained with sample balancing strategies adopted, we found that, for each threshold, samples with large vs. small SE changes could be successfully distinguished based on the non-cycloplegic data using all four machine learning models. The performances of different algorithms were generally similar, with the EEC model performed slightly better. When comparing the results across different thresholds, we found that the AUC increased gradually with the increase of threshold: AUC were about 0.762–0.807 when threshold value was 0.25D, 0.789–0.822 when threshold value was 0.50D, and 0.793–0.833 when threshold value was 0.75D.

### Prediction of cycloplegic refractive state

**Figure 3:**
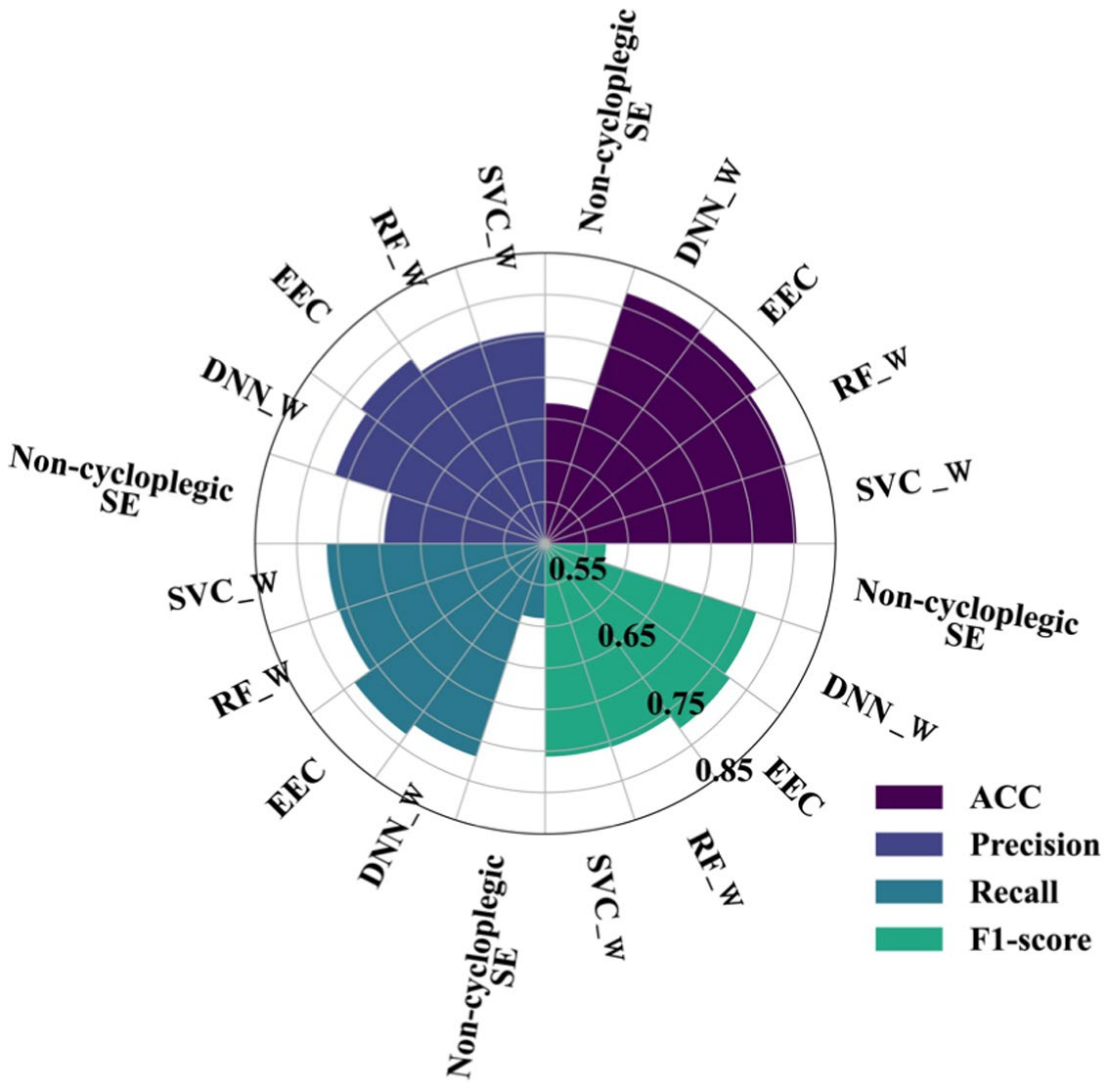
Performances of four machine learning algorithms and directly using non-cycloplegic SE for three- class classification. (SVC_W, Support Vector Machine with balance method; RF_W, Random Forest with balance method; DNN_W, Deep Neural Network with balance method; EEC, Easy Ensemble Classifier; ACC, accuracy; F1-score: balanced F Score).

The proportion of the three refractive states defined using cycloplegic SE were 42.58, 21.98 and 35.44% for myopia, emmetropia, and hyperopia, respectively, in the training set, and 48.37, 23.57 and 28.06%, respectively, in the test sets. The performances of the three-way classifications obtained using four machine learning algorithms with sample balancing strategies, together with the performances obtained directly using non-cycloplegic SE (the control condition) were summarized in online Supplementary Table S3 and Figure 3. The performances of machine learning algorithms without sample balancing strategies are shown in online Supplementary Table S4 and online Supplementary Figure S3 for contrast. The results showed that the classification performances of the four machine learning algorithms were similar (ACC ranging from 80.3 to 81.7%, precision ranging from 75.5 to 77.4%, recall rates ranging from 76.1 to 78.4%, F1 scores ranging from 0.757 to 0.775) and were much higher than those obtained directly from non-cycloplegic SE (ACC 66.8%, precision 69.4%, recall rates 59.0%, F1 scores 0.573).

Their corresponding confusion matrices with and without the sample balancing strategies are shown in online Supplementary Figures S4, S5, respectively. All confusion matrices obtained from the four machine learning models showed a clear diagonal structure (i.e., higher values on the diagonal), indicating successful classifications for each of the three classes, except the confusion matrix obtained directly from non-cycloplegic SE which showed a clear prediction bias toward myopia. Sensitivity and specificities for identifying myopia, emmetropia and hyperopia alone was shown in online Supplementary Table S5.

The proportions of the three refractive states defined using cycloplegic SE, non-cycloplegic SE, and obtained from the four machine learning algorithms predictions are shown in online Supplementary Figure S6 (the corresponding results obtained without the sample balancing strategies are shown in online Supplementary Figure S7). It clearly shows that non-cycloplegic SE strongly overestimated myopia and underestimated hyperopia, whereas the proportions predicted by the machine learning models were much closer to the cycloplegic proportions.

### Prediction of cycloplegic refractive error

The results of the machine learning algorithms in predicting the cycloplegic refractive error are summarized in online Supplementary Table S6. The prediction performances of the four machine learning models were similar (*r*^2^ ranging from 0.899 to 0.927, MSE ranging from 0.250 to 0.380, MAE ranging from 0.372 to 0.436, *r* ranging from 0.943 to 0.963) and much better than the non-cycloplegic SE estimates (*r*^2^ = 0.723, MSE = 0.942, MAE = 0.702, *r* = 0.922). Among the four models, AdaBoost regression model exhibited the best overall prediction performance, with *r*^2^ = 0.927, MSE = 0.250, MAE = 0.372, *r* = 0.963.

**Figure 4:**
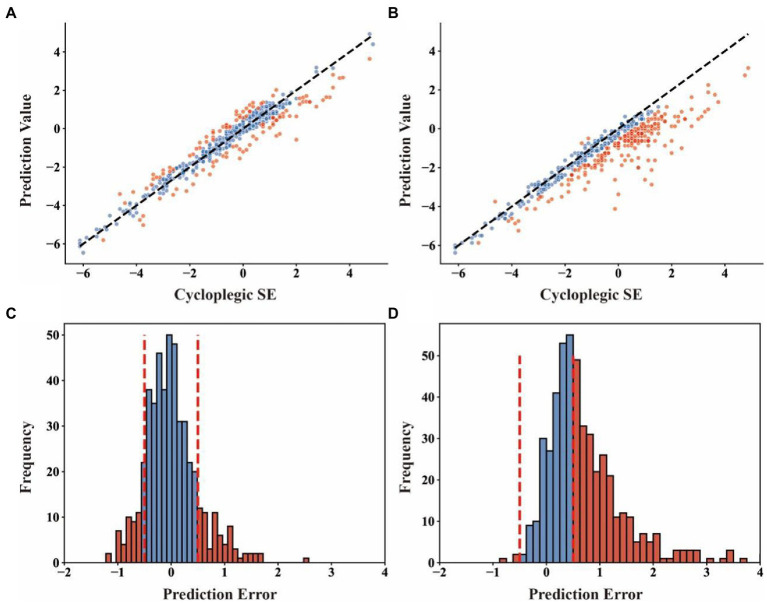
The scatter plot and distribution of prediction error of Adaboost regression model and directly using non-cycloplegic SE for the prediction of cycloplegic SE. **(A)**, the scatter plot with cycloplegic SE as the x-coordinate and predicted values from Adaboost regression model as the y-coordinate; **(B)**, the scatter plot with cycloplegic SE as the x-coordinates and predicted values directly using non-cycloplegic SE as the y-coordinates; **(C)**, the distribution of prediction errors by Adaboost regression model; **(D)**, the distribution of prediction errors by directly using non-cycloplegic SE. The red dots in **(A)** and **(B)** indicate the samples with prediction errors greater than 0.50D, and the vertical dashed lines in **(C)** and **(D)** indicate the boundary of the prediction error at 0.05D.

The scatter plot for the relationship between predicted refractive error by Adaboost regression model and actual cycloplegic SE values is shown in Figure 4A, and the scatter plot for the relationship between non-cycloplegic SE and actual cycloplegic SE is shown in Figure 4B.

To assess the clinical value of the machine-learning-model predictions of the refractive error, we defined clinically significantly inaccurate prediction as a bias greater than or equal to 0.50D compared with cycloplegic refractive error. We found that the percentage of the clinically inaccurate samples predicted by Adaboost regression model (24.8%) was much smaller than that by non-cycloplegic SE estimates (54.3%) (Figures 4C,D). matched samples t-test showed that there was no significant difference between the mean predicted SE by machine learning models and the mean cycloplegic SE (*p* = 0.169 for SVR, *p* = 0.153 for RFR, *p* = 0.533 for ABR, for DNN *p* = 0.227), whereas the difference between the mean non-cycloplegic SE and the mean cycloplegic SE was highly significant (*p* < 0.001).

### Weight analysis

**Figure 5:**
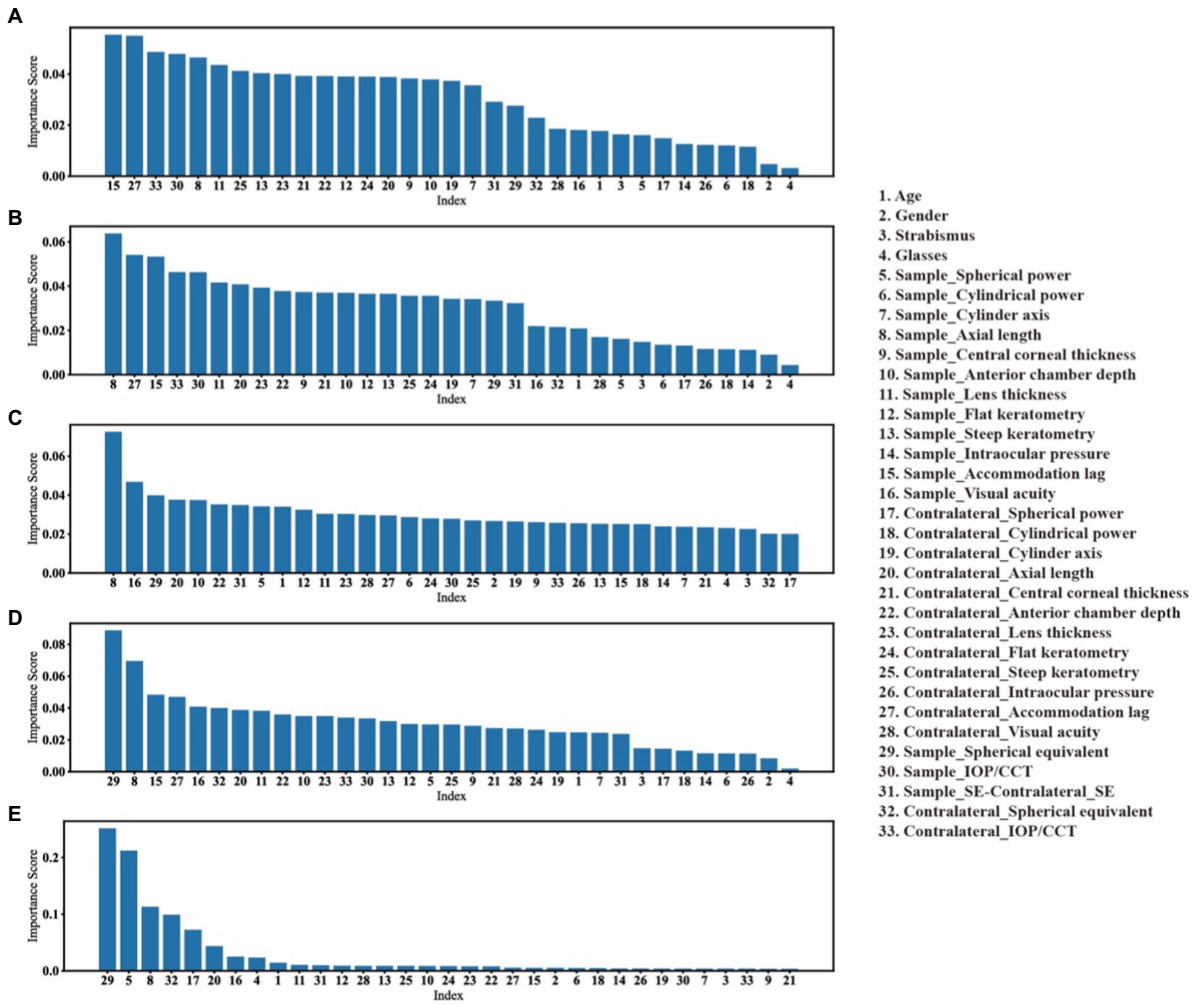
The features’ importance in each prediction task. We take the reduction in impurity as the importance of that feature. **(A–C)**, the features importance of EEC model in predicting the need for cycloplegia with threshold is 0.25D, 0.5D and 0.75D, respectively. **(D)**, the features importance of EEC model in predicting the cycloplegic refractive states. **(E)**, the features importance of ABR model in predicting the values of cycloplegic SE.

To further interpret machine learning models, we explored the importance of each feature in different prediction tasks, as shown in Figure 5. As the tree-based models (i.e., EEC and ABR) performed the best overall in all three prediction tasks, we used the tree-based models to measure the importance of a feature using the importance score that is calculated as the impurity decrease when using a feature in split of a tree node.

It was found that in the task of predicting the need for cycloplegia, different features had relatively similar weights under each threshold for defining large vs. small differences in SE before and after cycloplegia, but the ranking order of features varied across different thresholds.

In the refractive status and refractive error prediction tasks, the SE, Spherical power and AL were among the top three features with the greatest importance, which is consistent with the fact that non-cycloplegic SE is often used to predict refractive status and refractive error in clinical work. Especially in the cycloplegic refractive error prediction model, these three features (SE, Spherical power and AL) were able to reduce the impurity by about 90%.

## Discussion

To resolve the conflict between the necessity of cycloplegic refractive examination and the difficulty of performing cycloplegia in children and adolescents in practice, we applied a variety of commonly used machine learning algorithms to predict cycloplegic data based on non-cycloplegic data from a large dataset of Chinese school-age children. The predictions of the cycloplegic data were made at three different levels to meet the needs in different scenarios: prediction of the need for cycloplegia, prediction of the refractive status, and prediction of the refractive error. Our results showed successful predictions at all three levels, demonstrating the promising potential and practical value of predicting cycloplegic data using machine learning techniques based on non-cycloplegic data in clinical applications and epidemiological studies.

### Identify patients with the need for cycloplegia

Several studies have shown that in children and adolescents, the fluctuation in refractive diopter is affected by many factors under non-cycloplegia condition (1, 2, 7, 11, 14–16), and the degree of such fluctuation varies greatly across individuals (33, 34). Clinically, recognizing when it is appropriate to forgo cycloplegia and when it is necessary to conduct cycloplegia for accurate refractive measurement is very useful for avoiding unnecessary cycloplegia. Simply thresholding in age and the state of ametropia cannot accurately identify the target patients with the need for cycloplegia (35, 36).

We obtained 30 parameters through clinical optometry routine examination under non-cycloplegia combined with machine learning analyses to predict the need for cycloplegia for the first level prediction. Identifying “significant SE change” is an important basis for cycloplegia in clinical decision. In this study, three thresholds were set for predicting SE changes before and after cycloplegia, which were >0.25D, >0.50D and >0.75D. For predicting the target persons of SE change >0.50D, the performance of EEC model can reach an AUC of 0.822, with a specificity of 76.2% and a sensitivity of 74.4%. If the threshold of >0.75D was used to define the target persons, our EEC model can reach an AUC of 0.833, with a specificity of 78.5% and a sensitivity of 73.9%. We also tested the efficiency of the model at the threshold of >0.25D, which might not be of great clinical significance for a difference in SE between before and after cycloplegia, the EEC model still can reach an AUC of 0.807, with a specificity of 77.6% and a sensitivity of 69.5%. In the actual application of model prediction, we will adjust the model to keep the model high sensitivity, which ensures that patients who need cycloplegia are not missed. According to ROC curve, in the EEC model 0.50D threshold, when the sensitivity is set to 0.90, the specificity is 0.57, which means that more than half of the patients who do not need cycloplegic can be excluded from unnecessary cycloplegic operations when the patients who need cycloplegic are basically identified.

In addition, the weight analyses showed relatively similar importance scores for different features under each threshold for defining large vs. small differences in SE before and after cycloplegia, and with different ranking orders across different thresholds, suggesting that most features contributed to this binary prediction task.

Our results showed that the developed machine learning models could successfully identify the target patients and thus help avoid unnecessary cycloplegias, which may be valuable in clinical practice to reduce cycloplegia workload, or helpful to the optometrists in optical shops.

### Improve the accuracy of refractive state assessment and refractive error measurement

It is known that assessing the refractive state using non-cycloplegic refractive data directly will lead to a myopic shift in the mean refractive error in school-age children (9, 10). Consistent with previous studies, such bias was also clearly observed in our study: 1301 (75.1%) eyes of hyperopia were wrongly assessed as emmetropic or even myopia and 535 (49.9%) eyes of emmetropic were wrongly assessed as myopia, thus an overestimation of myopia and emmetropia, and underestimation of hyperopia. Similarly, the mean refractive error changed from −0.51 ± 1.81 D to −1.13 ± 1.58 D under the non-cycloplegia condition with a mean difference of 0.63 ± 0.69 D toward myopia.

Sankaridurg et al. established a linear regression model for children and adolescents aged 4–15 years, and predicted cycloplegic SE by age, uncorrected vision acuity, and non-cycloplegic SE. The prediction model R2 was 0.91, and 77% of participants were correctly predicted in refractive state, but the independent validation set was not used to test the effectiveness of the model (13).

In our study, the machine learning models predicted the distribution of refractive status with an accuracy of 81.7%, and for the Adaboost model of cycloplegic refractive error prediction, 75.2% of the predicted SE had a prediction error less than 0.50D. Such predictive power was much higher than direct non-cycloplegic SE estimates. At the same time, a t-test showed no significant difference in the means between the predicted SE of the model and the real SE. In addition, three easy-to-obtain parameters SE, Spherical Power and Axial Length were found to play an important role in the prediction. These results are reasonable, because these features indicate the pre-cycloplegia refractive result of the patient directly and the outcomes of pre-cycloplegic refraction are highly correlated with post-cycloplegic outcomes (2, 7). We speculate that the model also starts with the pre-cycloplegia refractive results, and adjust the results by some features related to ciliary muscle tension to obtain the final estimation.

The results of our present study suggest that, in school-based vision screening and epidemiological studies where cycloplegia may be considered impractical, predicting cycloplegic refractive error and refractive status by machine learning models based on noncycloplegic data at individual level may be an effective way. Especially, such improvement of the refractive state assessment and refractive error measurement provides an accurate estimation of the distributions of refractive status and refractive diopter at the population level, and thus has great value for epidemiological studies.

## Limitations

There were several limitations in the present study. First, although missing data was a common problem in studies with large dataset and data imputation was adopted to remedy this problem in the present study, the missing data in our dataset may still have an impact on the performance of our machine learning models. Second, some ocular parameter used to build the machine learning models might not be easily available in practice, which would limit the applicability of the developed models in the present study. For example, the accommodation lag data in this study were obtained by open field autorefraction. In future studies, this measure may be replaced by dynamic retinoscopy that is a more convenient and widely available technique. Finally, although the sample size and features set in the present study was large compared to most previous studies (In this study, in order to expand the sample size, both eyes of a person are included in the data set, and there is a correlation between the eyes, which may have a certain impact on the robustness of the model), more data may be needed for developing machine learning models with higher prediction accuracies.

## Conclusion

The machine learning algorithm can be used to estimate the demand for cycloplegia, the cycloplegia refractive error and the refractive status using the non-cycloplegia parameters. It is of application value in clinical work and epidemiological research.

## Data availability statement

The original contributions presented in the study are included in the article/Supplementary material, further inquiries can be directed to the corresponding authors.

## Ethics statement

The studies involving human participants were reviewed and approved by the human ethics committees at the Tianjin Medical University Eye Hospital. Written informed consent to participate in this study was provided by the participants' legal guardian/next of kin.

## Author contributions

BD, QW, and YL: collection and assembly of data, data analysis and interpretation, and manuscript writing. NJ and HR: collection of data. XW and HN: provision of study material. LG, ML, and RW: conception and design, financial support, manuscript writing, and final approval. All authors contributed to the article and approved the submitted version.

## Funding

This study was funded by the Social Science Major Project of Tianjin Municipal Education Commission (2020JWZD20 and 2022JWZD23), National Natural Science Foundation of China, Grant/Award number: 82070929, Tianjin Clinical Key Discipline Project (TJLCZDXKQ012) and (TJLCZDXKM007), and Tianjin Key Medical Discipline (Specialty) Construction Project (TJYXZDXK-037A). The funders had no role in study design, data collection and analysis, decision to publish, or preparation of the manuscript.

## Conflict of interest

The authors declare that the research was conducted in the absence of any commercial or financial relationships that could be construed as a potential conflict of interest.

## Publisher’s note

All claims expressed in this article are solely those of the authors and do not necessarily represent those of their affiliated organizations, or those of the publisher, the editors and the reviewers. Any product that may be evaluated in this article, or claim that may be made by its manufacturer, is not guaranteed or endorsed by the publisher.

## Supplementary material

The Supplementary material for this article can be found online at: https://www.frontiersin.org/articles/10.3389/fpubh.2023.1096330/full#supplementary-material
