# Supplementary material for: Prediction of spherical equivalent difference before and after cycloplegia in school-age children with machine learning algorithms
Source: Front Public Health. 2023 Apr 11;11:1096330. doi: 10.3389/fpubh.2023.1096330 (PMC10126339; doi:10.3389/fpubh.2023.1096330)
Supplement: Supplementary file 1 [file Table_1.DOCX]

**Table S1. Performances of four machine learning models for binary classification of each class defining threshold.**

| **Threshold** | **Model** | **Accuracy(%)** | **Sensitivity(%)** | **Specificity(%)** | **AUC** |
| --- | --- | --- | --- | --- | --- |
| 0.25D | SVC_W | 70.3% | 68.9% | 73.3% | 0.786 |
|  | RF_W | 68.5% | 69.5% | 66.5% | 0.762 |
|  | DNN_W | 72.2% | 69.5% | 77.6% | 0.807 |
|  | EEC | 74.0% | 78.2% | 65.2% | 0.796 |
|  |  |  |  |  |  |
| 0.5D | SVC_W | 74.2% | 71.2% | 76.6% | 0.800 |
|  | RF_W | 74.4% | 69.9% | 78.0% | 0.789 |
|  | DNN_W | 75.4% | 74.4% | 76.2% | 0.822 |
|  | EEC | 74.2% | 71.2% | 76.6% | 0.812 |
|  |  |  |  |  |  |
| 0.75D | SVC_W | 75.8% | 68.0% | 79.4% | 0.829 |
|  | RF_W | 75.2% | 69.3% | 77.9% | 0.793 |
|  | DNN_W | 77.0% | 73.9% | 78.5% | 0.833 |
|  | EEC | 77.0% | 68.6% | 80.8% | 0.805 |

(SVC_W: Support Vector Machine with balance method; RF_W: Random Forest with balance method; DNN_W: Deep Neural Network with balance method; EEC: Easy Ensemble Classifier; AUC: area under the ROC curve)

**Table S2. Performances of machine learning algorithms without sample balancing strategies for binary classification of each class defining threshold.**

| **Threshold** | **Model** | **Accuracy(%)** | **Sensitivity(%)** | **Specificity(%)** | **AUC** |
| --- | --- | --- | --- | --- | --- |
| 0.25D | SVC | 69.5% | 77.9% | 52.1% | 74.8% |
|  | RF | 72.7% | 83.9% | 49.6% | 76.4% |
|  | DNN | 73.1% | 82.4% | 54.0% | 78.3% |
|  |  |  |  |  |  |
| 0.5D | SVC | 74.6% | 70.7% | 77.6% | 80.8% |
|  | RF | 73.9% | 67.5% | 79.1% | 78.8% |
|  | DNN | 76.4% | 71.6% | 80.2% | 82.0% |
|  |  |  |  |  |  |
| 0.75D | SVC | 79.8% | 58.8% | 89.4% | 83.7% |
|  | RF | 77.0% | 47.0% | 90.6% | 79.4% |
|  | DNN | 79.6% | 60.1% | 88.5% | 81.9% |

(SVC: Support Vector Machine without balance method; RF: Random Forest without balance method; DNN: Deep Neural Network without balance method; AUC: area under the ROC curve)

**Table S3. Performances of four machine learning algorithms and directly using non-cycloplegic SE for three-class classification.**

|  | **Accuracy(%)** | **Precision(%)** | **Recall(%)** | **F1 score** |
| --- | --- | --- | --- | --- |
| SVC_W | 80.3% | 75.5% | 76.3% | 0.757 |
| RF_W | 80.5% | 75.5% | 76.1% | 0.757 |
| DNN_W | 81.7% | 76.6% | 77.0% | 0.768 |
| EEC | 81.5% | 77.4% | 78.4% | 0.775 |
| Non-cycloplegic SE | 59.2% | 66.4% | 54.1% | 0.503 |

(SVC_W: Support Vector Machine with balance method; RF_W: Random Forest with balance method; DNN_W: Deep Neural Network with balance method; EEC: Easy Ensemble Classifier)

**Table S4. Performance of machine learning algorithms without sample balancing strategies for three-class classification.**

|  | **Accuracy(%)** | **Precision(%)** | **Recall(%)** | **F1 score** |
| --- | --- | --- | --- | --- |
| SVC | 79.3% | 72.7% | 72.4% | 0.724 |
| RF | 80.7% | 74.5% | 73.5% | 0.736 |
| DNN | 81.1% | 75.1% | 74.7% | 0.748 |

(SVC: Support Vector Machine without balance method; RF: Random Forest without balance method; DNN: Deep Neural Network without balance method EEC: Easy Ensemble Classifier)

**Table S5. Sensitivity and specificities for identifying myopia, emmetropia and hyperopia.**

|  |  | Myopia | Emmetropia | Hyperopia |
| --- | --- | --- | --- | --- |
| SVC | Recall | 88.2% | 60.6% | 80.0% |
|  | Specificity | 72.8% | 84.9% | 80.4% |
|  |  |  |  |  |
| RF | Recall | 89.1% | 58.5% | 80.6% |
|  | Specificity | 72.4% | 85.7% | 80.4% |
|  |  |  |  |  |
| EEC | Recall | 88.2% | 67.0% | 80.0% |
|  | Specificity | 75.2% | 84.9% | 82.2% |
|  |  |  |  |  |
| DNN | Recall | 90.3% | 57.4% | 83.1% |
|  | Specificity | 73.6% | 87.4% | 81.0% |

(SVC: Support Vector Machine without balance method; RF: Random Forest without balance method; DNN: Deep Neural Network without balance method; EEC: Easy Ensemble Classifier)

**Table S6. Performances of four machine learning algorithms and directly using non-cycloplegic SE for the prediction of cycloplegic SE.**

|  | **r^2^** | **MSE** | **MAE** | **r** |
| --- | --- | --- | --- | --- |
| SVR | 0.926 | 0.252 | 0.387 | 0.963 |
| RFR | 0.889 | 0.380 | 0.436 | 0.943 |
| ABR | 0.927 | 0.250 | 0.372 | 0.963 |
| DNN | 0.923 | 0.263 | 0.399 | 0.961 |
| Non-cycloplegic SE | 0.723 | 0.942 | 0.702 | 0.922 |

(SVR: Support Vector Regression; RFR: Random Forest Regression; ABR: AdaBoost Regression; DNN: Deep Neural Network)

**
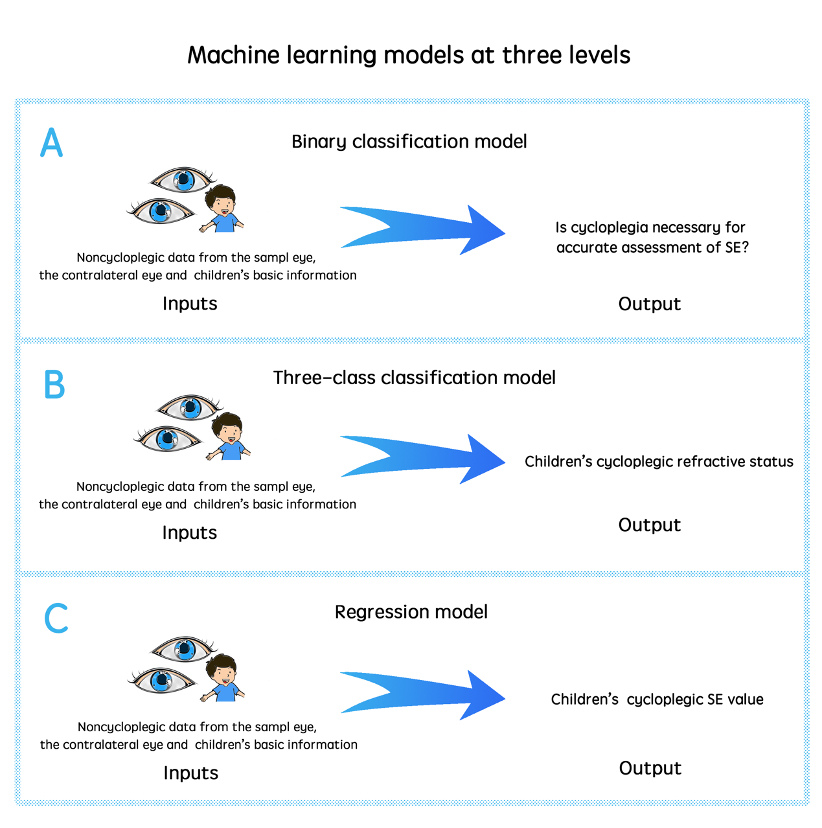
**

**Figure S1. Three levels prediction models.** (A) Using binary classification models with non-cycloplegic data to predict whether a participant needs cycloplegia. (B) Using three-way classification models with non- cycloplegic data to predict whether a participant is myopia, emmetropia or hyperopia. (C) Using regression models with non-cycloplegic data to predict participant’s refractive error.


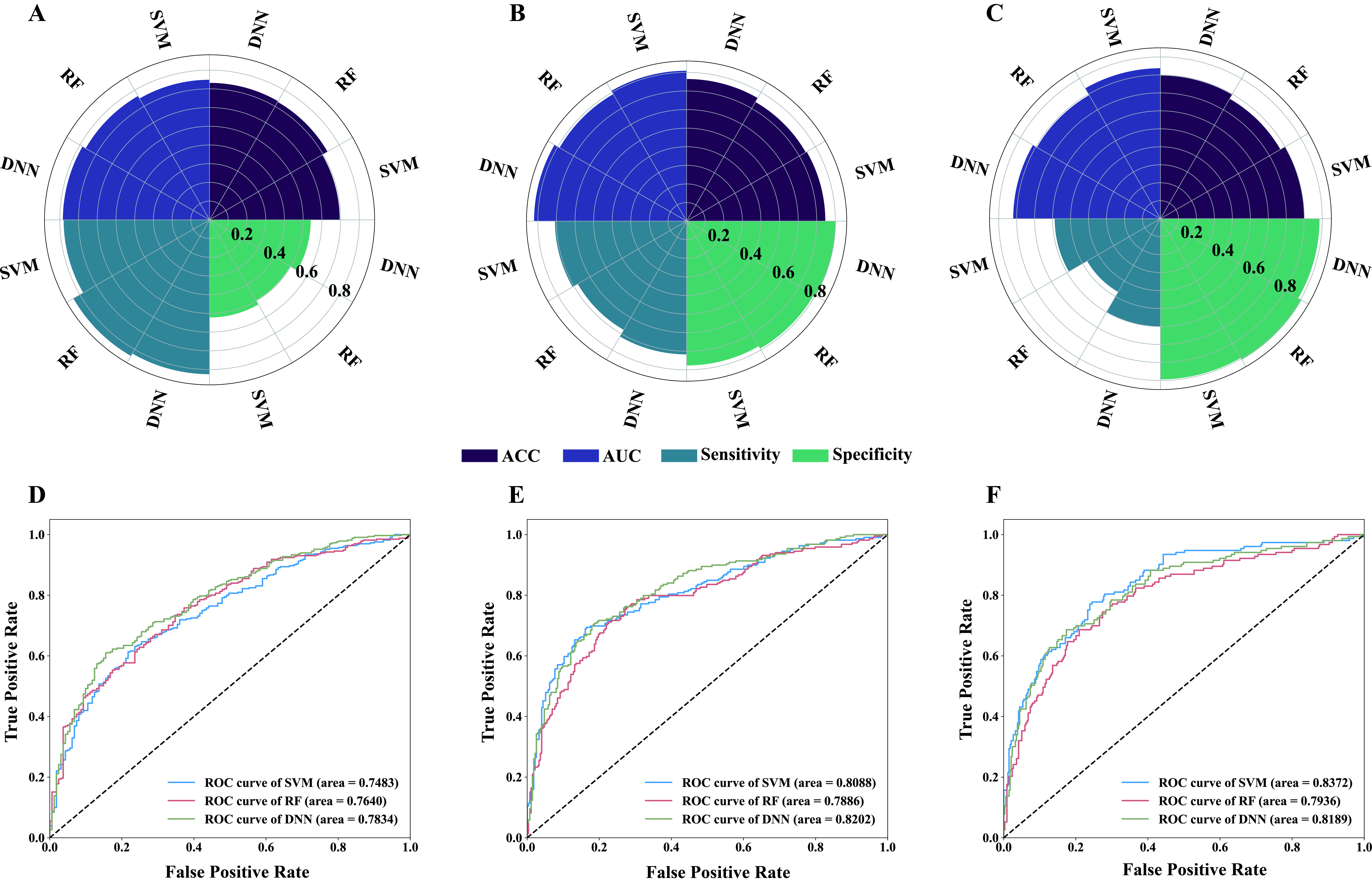


**Figure S2 Performance of machine learning algorithms without sample balancing strategies for binary classification of each class defining threshold.** (A, D), Threshold 0.25D, diagnostic values and ROC analysis of the four different predictive model; (B, E), Threshold 0.5D, diagnostic values and ROC analysis of the four different predictive model; (C, F), Threshold 0.75D, diagnostic values and ROC analysis of the four different predictive model. (SVC: Support Vector Machine without balance method; RF: Random Forest without balance method; DNN: Deep Neural Network without balance method; AUC: area under the ROC curve)


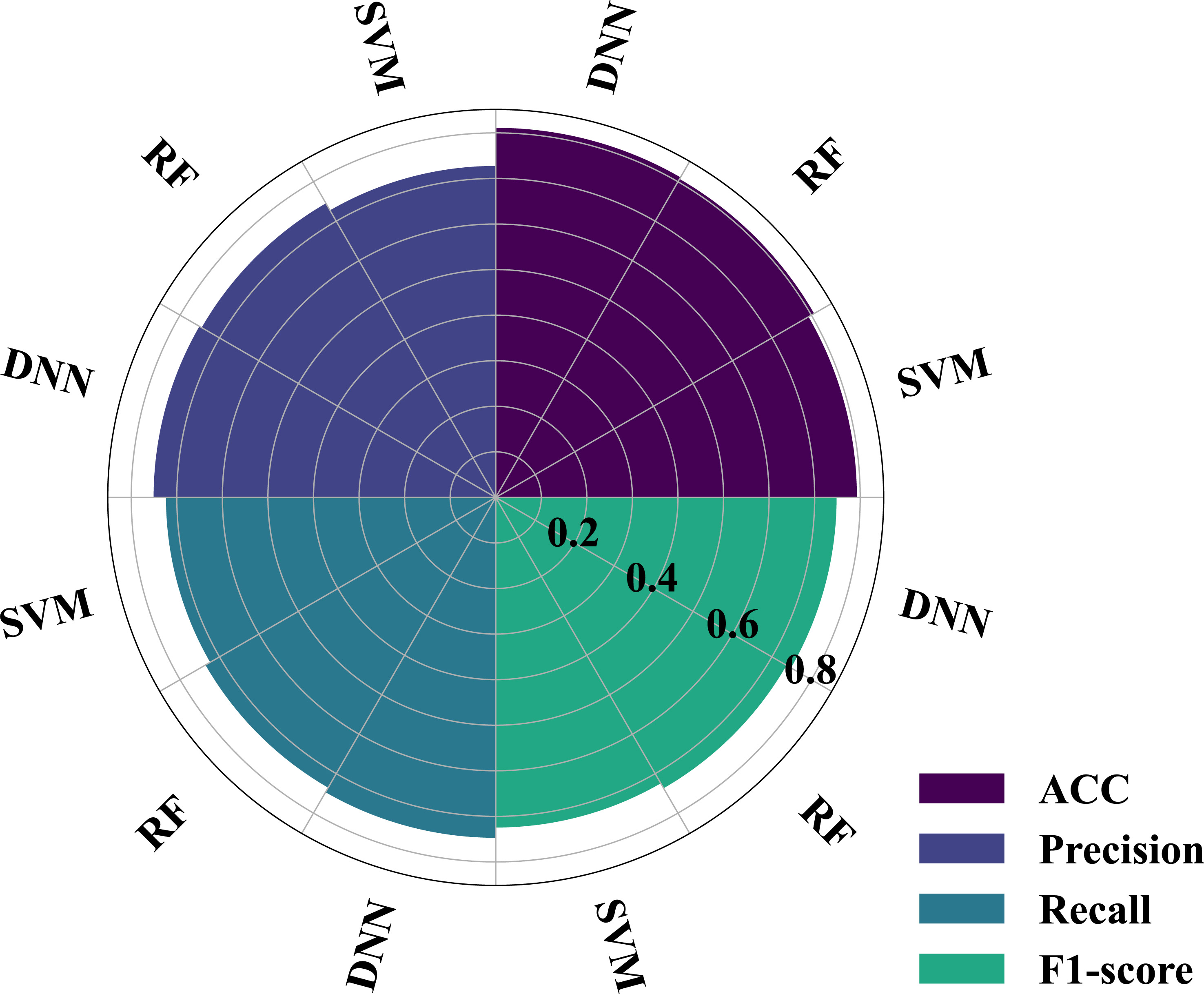


**Figure S3 Performances of four machine learning algorithms without sample strategies for three-class classification.** (SVM: Support Vector Machine without balance method; RF: Random Forest without balance method; DNN: Deep Neural Network without balance method)

**
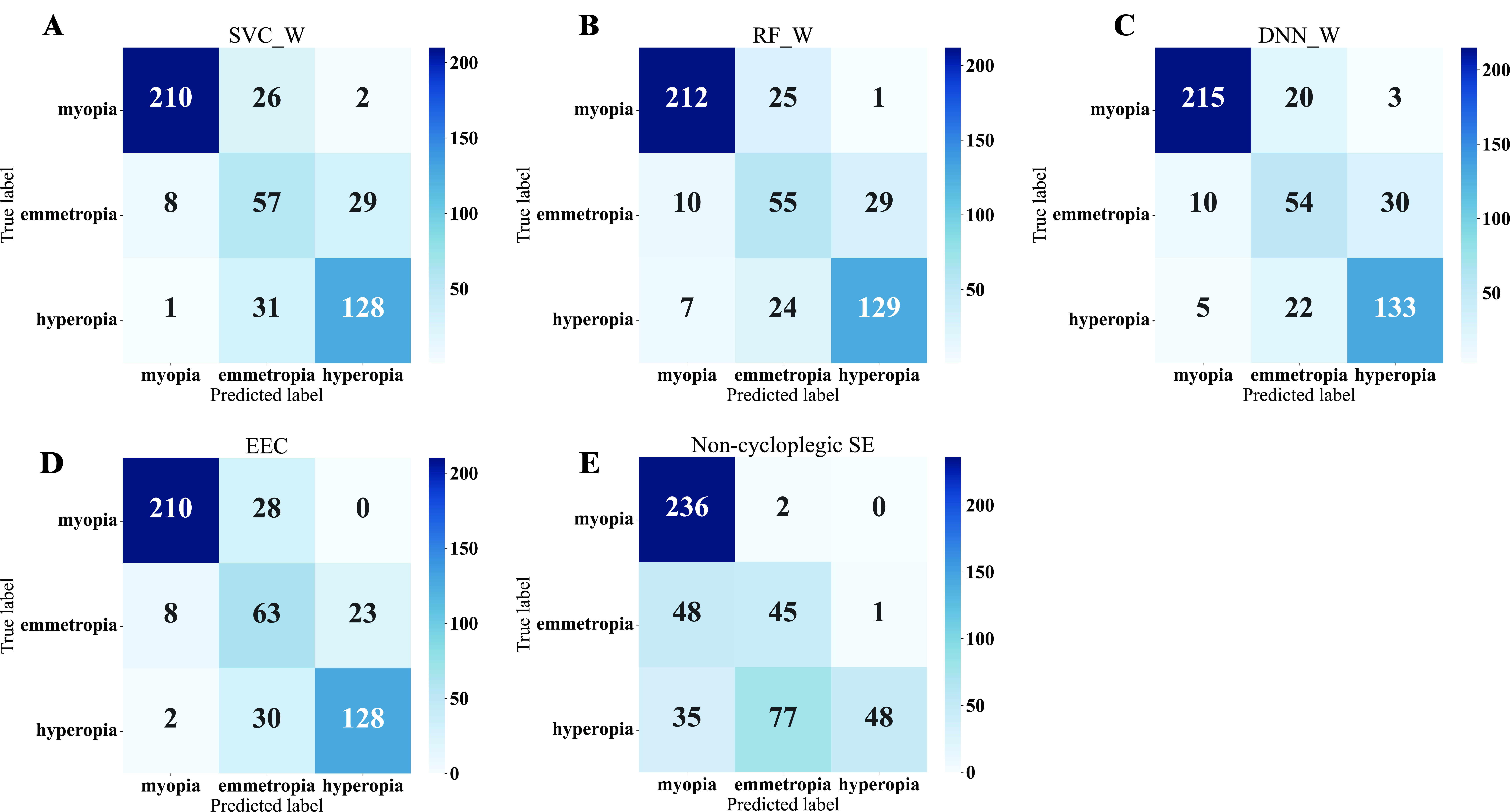
**

**Figure S4. Confusion matrices of four machine learning algorithms and directly using non-cycloplegic SE for three-class classification.**

(A) Confusion matrices for SVC_W algorithms. (B) Confusion matrices for RF_W algorithms. (C) Confusion matrices for DNN_W algorithms. (D) Confusion matrices for EEC algorithms. (E) Confusion matrices for directly using non-cycloplegic SE. (SVM_W: Support Vector Machine with balance method; RF_W: Random Forest with balance method; DNN_W: Deep Neural Network with balance method; EEC: Easy Ensemble Classifier)


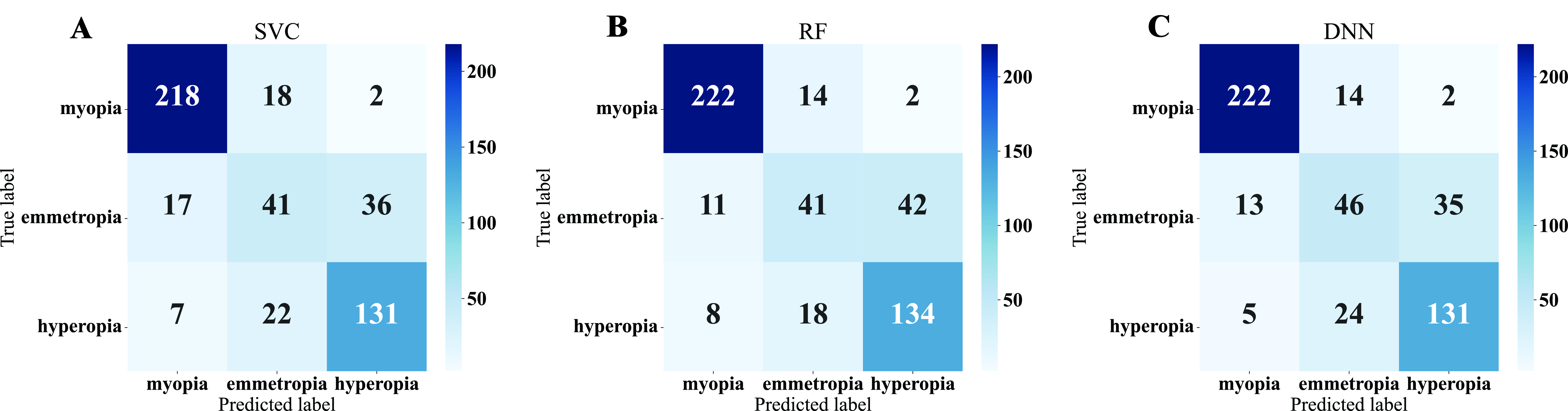


**Figure S5 Confusion matrices of machine learning algorithms without sample strategies for three-class classification.** (A) Confusion matrices for SVC algorithms. (B) Confusion matrices for DNN algorithms. (C) Confusion matrices for RF algorithms. (SVC: Support Vector Machine without balance method; RF: Random Forest without balance method; DNN: Deep Neural Network without balance method)


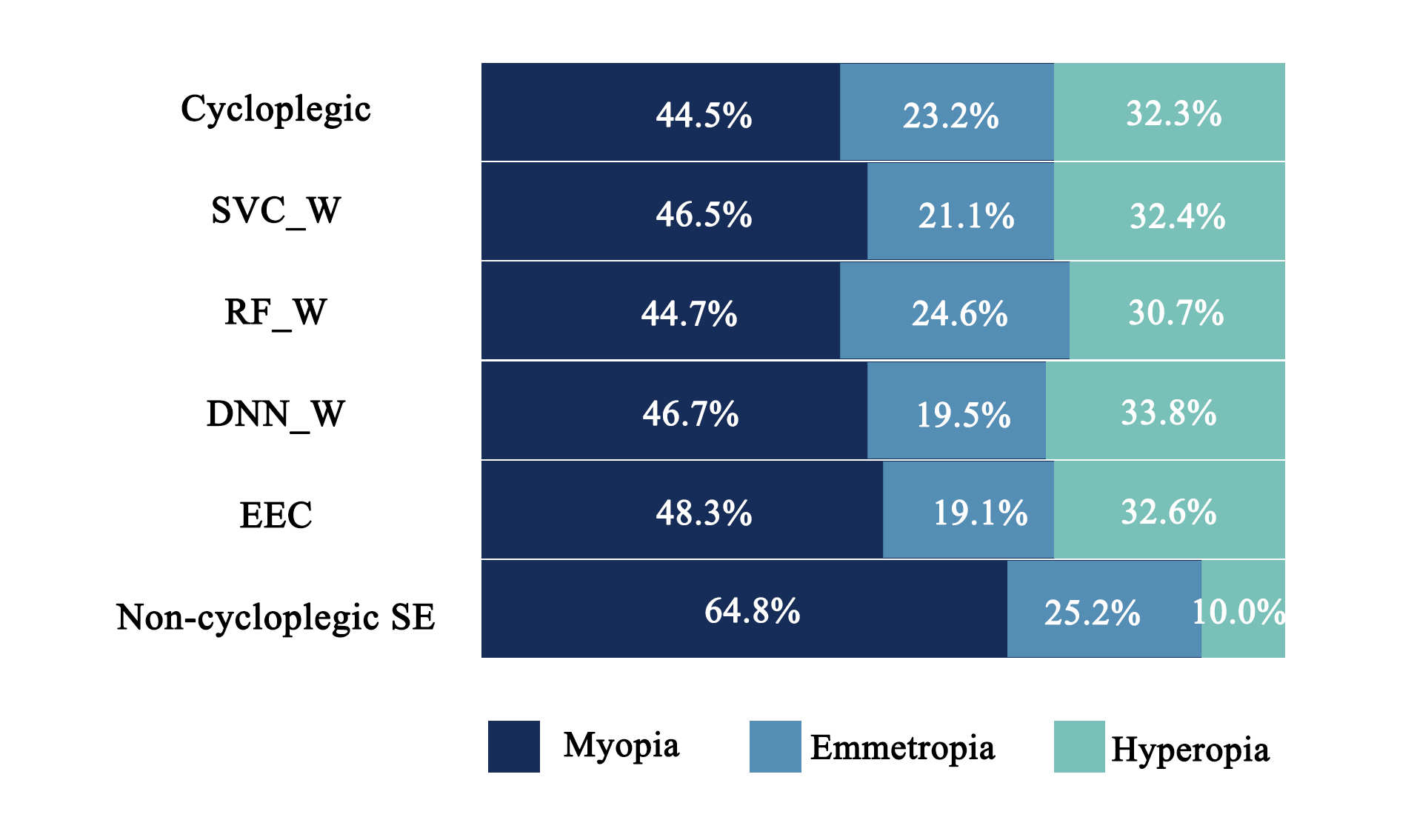


**Figure S6. The proportions of the myopia, emmetropia and hyperopia defined using cycloplegic SE, non-cycloplegic SE, and obtained from the four machine learning algorithms.** (SVC_W: Support Vector Machine with balance method; RF_W: Random Forest with balance method; DNN_W: Deep Neural Network with balance method; EEC: Easy Ensemble Classifier)


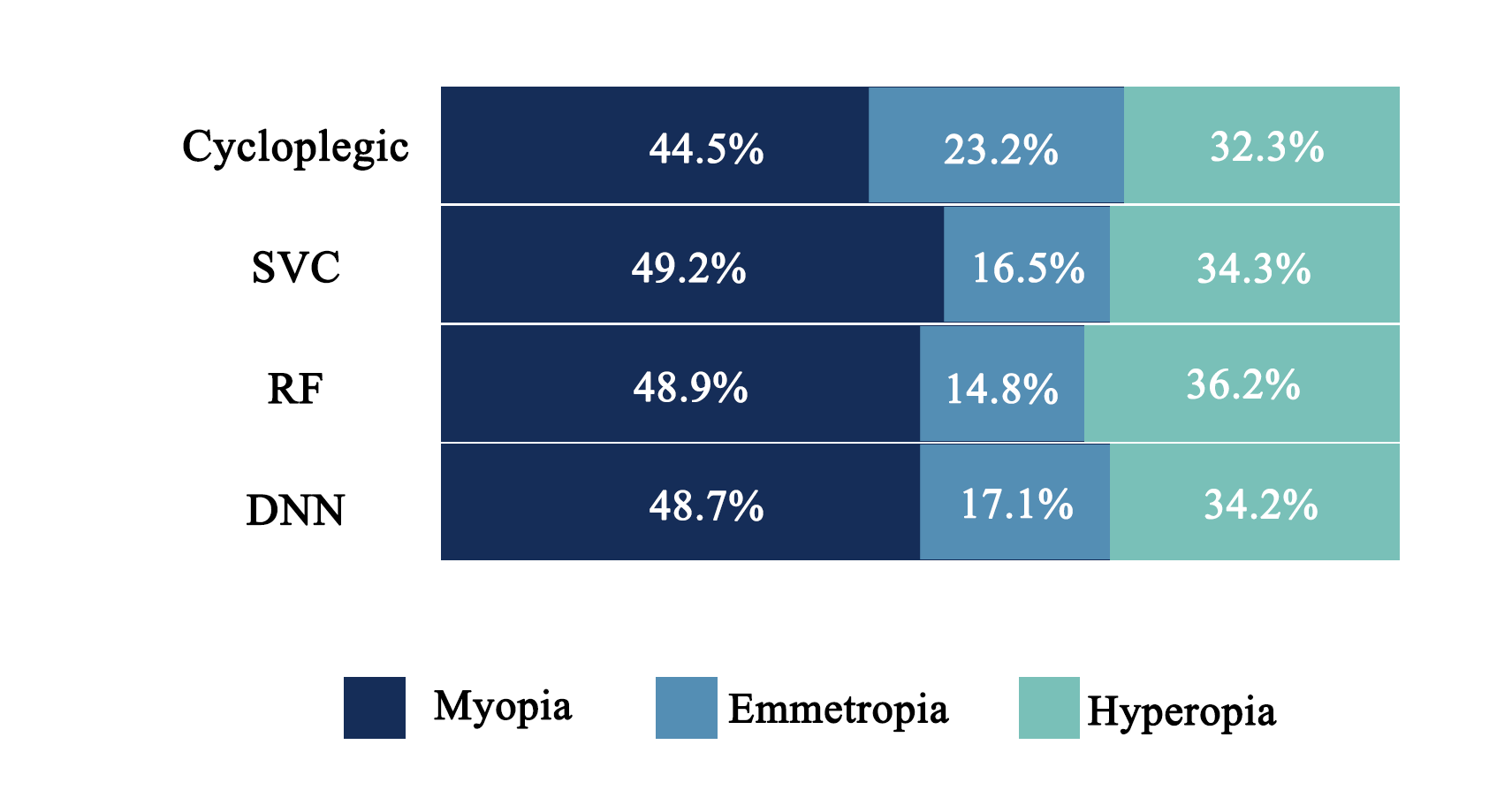


**Figure S7 The proportions of the myopia, emmetropia and hyperopia defined using cycloplegic SE and obtained from the four machine learning algorithms without sample balancing strategies.** (SVM: Support Vector Machine without balance method; RF: Random Forest without balance method; DNN: Deep Neural Network without balance method)
